# LAMP-Based 4-Channel Microfluidic Chip for POCT Detection of Influenza A H1N1, H3N2, and Influenza B Victoria Viruses

**DOI:** 10.3390/bios15080506

**Published:** 2025-08-04

**Authors:** Xue Zhao, Jiale Gao, Yijing Gu, Zheng Teng, Xi Zhang, Huanyu Wu, Xin Chen, Min Chen, Jilie Kong

**Affiliations:** 1 Virus Testing Laboratory, Pathogen Testing Center, Shanghai Municipal Center for Disease Control and Prevention, Shanghai 201100, China; 2 School of Life Science and Technology, Tongji University, Shanghai 200092, China; 3 Chemistry Department, Fudan University, Shanghai 200433, China

**Keywords:** influenza virus, loop-mediated isothermal amplification, microfluidic chip, nucleic acid detection

## Abstract

**Background**: Influenza viruses are major pathogens responsible for respiratory infections and pose significant risks to densely populated urban areas. RT-qPCR has made substantial contributions in controlling virus transmission during previous COVID-19 epidemics, but it faces challenges in terms of detection time for large sample sizes and susceptibility to nucleic acid contamination. **Methods**: Our study designed loop-mediated isothermal amplification primers for three common influenza viruses: A/H3N2, A/H1N1, and B/Victoria, and utilized a 4-channel microfluidic chip to achieve simultaneous detection. The chip initiates amplification by centrifugation and allows testing of up to eight samples at a time. **Results**: By creating a closed amplification system in the microfluidic chip, aerosol-induced nucleic acid contamination can be prevented through physically isolating the reaction from the operating environment. The chip can specifically detect A/H1N1, A/H3N2, and B/Victoria and has no signal for other common respiratory viruses. The testing process can be completed within 1 h and can be sensitive to viral RNA at concentrations as low as 10^−3^ ng/μL for A/H1N1 and A/H3N2 and 10^−1^ ng/μL for B/Victori. A total of 296 virus swab samples were further analyzed using the microfluidic chip method and compared with the classical qPCR method, which resulted in high consistency. **Conclusions**: Our chip enables faster detection of influenza virus and avoids nucleic acid contamination, which is beneficial for POCT establishment and has lower requirements for the operating environment.

## 1. Introduction

Influenza viruses are one of the major pathogens causing respiratory infections worldwide [1,2,3]. Among them, Influenza A virus and Influenza B virus are the primary types responsible for seasonal flu outbreaks [4]. Influenza A viruses can be divided into various subtypes based on differences in hemagglutinin and neuraminidase [5], such as A/H1N1 and A/H3N2. Influenza B viruses are classified into two lineages: B/Victoria and B/Yamagata. Both of the two lineages of Influenza B viruses are recognized as circulating subtypes of seasonal influenza. Currently, no Yamagata type has been detected and reported in the world, and the main prevalent strain is the Victoria type [6]. These influenza viruses can be highly variable because their antigenic properties have the ability to drift or shift, which contribute to their quick evolution [7]. Rapidly changing surface antigens and possible genetic mutations are significant challenges for vaccine design and disease control efforts [8]. A/H1N1, A/H3N2, and B/Victoria are widely spread among populations and can cause a range of clinical manifestations, from mild respiratory symptoms to severe pneumonia and even death [9]. The A/H1N1 virus caused a global pandemic in 2009; it spread rapidly and infected millions of people in more than 214 countries [10]. A/H3N2 is associated with severe flu seasons, but the vaccine is not very protective [11]. B/Victoria has received less attention in the past but appears to be increasing in potency and is associated with high morbidity and mortality [12].

Influenza surveillance data from Shanghai Municipal Center for Disease Control and Prevention indicate that since 2021 [13], A/H1N1pdm09, A/H3N2, and B/Victoria have alternated or co-circulated in different seasons. A/H1N1pdm09 is a new strain formed by the recombination of humans, poultry, and pigs [14]. After 2009, it replaced the old A/H1N1 virus and became the main strain prevalent in the human population. B/Victoria was the dominant strain during the winter–spring season of 2021–2022. In the winter–spring season of 2022–2023, all three subtypes co-circulated, with A/H1N1pdm09 and A/H3N2 being the predominant strains. In the summer of 2023, a small peak of A/H3N2 was observed [13]. During the winter–spring season of 2023–2024, all three subtypes again co-circulated, with A/H3N2 predominating in the early phase and B/Victoria taking dominance in the later phase. In the winter–spring season of 2024–2025, A/H1N1pdm09 emerged as the dominant circulating subtype.

During the COVID-19 pandemic, the qPCR method was widely used for viral nucleic acid detection, and the importance of testing speed in disease control became increasingly evident. As a relatively mature nucleic acid (NA) detection method, loop-mediated isothermal amplification (LAMP) has the ability to rapidly amplify NAs under constant temperature conditions [15]. LAMP employs four to six primers that can accurately identify target sequences, theoretically offering superior specificity compared to the qPCR method [16]. With the help of Bst DNA polymerase, LAMP can obtain a large amount of amplification products in a short time through a chain displacement reaction, and the entire reaction is completed at a constant temperature of 65 °C [17]. However, in extensive studies, LAMP has been found to be easily interfered with by environmental NA contamination, making it difficult to operate stably in high throughput detection lines filled with NA aerosols [18]. Additionally, the longer primers used in LAMP can potentially dimerize, which is often the cause of false-positive results [19], even when working in biosafety cabinets or on clean benches and following proper disinfection protocols.

Previous studies have focused on the problem of false positives in LAMP. Schneider et al. [20] established a mathematical model to identify false-positive results produced by LAMP, while Hardinge and Murray [21] used quenched fluorescent primers to reduce the occurrence of false positives. Subsequently, many microfluidic technologies were used to carry out LAMP reactions for pathogen detection [22,23,24]. They are mainly used to achieve multiple detection, integration, absolute quantification and other functions [25]. Among them, the centrifugal microfluidic chip utilizes rotation as the power source, offering high integration and ease of miniaturization [26]. It is also well-suited for high-throughput parallel processing and can accommodate various NA amplification methods [22]. Nguyen et al. [27] used a centrifugal microfluidic chip combined with RT-LAMP technology to detect four respiratory viruses, which took 1.5 h per test. Dong et al. [28] used RPA technology to detect different genes of the African swine fever virus in parallel, with high amplification efficiency and parallel processing capabilities.

We used a LAMP microfluidic chip suitable for POCT (Point-of-Care Testing) to detect three influenza viruses. By pre-encapsulating amplification reagents into microfluidic chips in a contamination-free environment, aerosol issues could be effectively addressed. The closed microfluidic chip uses low-cost polycarbonate as the main material, and the pre-processing can be quickly completed by loading samples and stickers. We employed RT-LAMP (reverse transcription loop-mediated isothermal amplification) reagents targeting RNA samples, combined with LAMP primers specific to A/H1N1 (the following refers to pdm09), A/H3N2, and B/Victoria. During the centrifugation step of the LAMP microfluidic chip, RT-LAMP reagents and primers were mixed to initiate the reaction. Significantly, at least 300 A/H1N1, A/H3N2, and B/Victoria samples from various regions in Shanghai at different years were collected for primer design and screening tests. The primer sets and LAMP microfluidic chip enable the detection of viral fragments at concentrations as low as 10^2^ copies/μL within 1 h, with excellent reproducibility. Compared to RT-qPCR, the LAMP microfluidic chip shows good consistency and holds potential for large-scale applications.

## 2. Materials and Methods

### 2.1. Primer Design

Nasal swab samples or virus cultures were collected from infected patients previously stored by the Shanghai Municipal Center for Disease Control and Prevention, along with other available sequencing results. The results included 120 cases of A/H1N1 (2009-present), 528 cases of A/H3N2 (2006-present), and 51 cases of B/Victoria (2023-present).

Nasal swab samples and virus cultures were processed using a magnetic bead-based Viral NA rapid extraction kit (SDK004, Jiangsu Bioperfectus Technologies Co., Ltd., Taizhou, China) with an automatic NA extractor (SNP-A, Jiangsu Bioperfectus Technologies Co., Ltd.). RNA sequences of all collected influenza samples were aligned using Mega (version 7.0). Supplementary Tables S1–S3 present the alignment results for A/H1N1, A/H3N2, and B/Victoria, respectively. Conserved regions of each influenza virus were selected for primer design.

LAMP primers were designed based on multiple targets from the conserved regions of A/H1N1, A/H3N2, and B/Victoria using the automatic judgment mode in Primer Explorer V5 (https://primerexplorer.eiken.co.jp/lampv5e/index.html (accessed on 28 July 2025)). Based on subsequent screening results, suitable inner and outer primers were selected for further design of loop primers.

### 2.2. Amplification and Detection Procedures of LAMP Microfluidic Chip

The LAMP microfluidic chip (KY0000002) and the corresponding detection instrument (MA3000) were manufactured by Shanghai Igenetec Diagnostics Co., Ltd. (Shanghai, China). Before starting amplification, the instrument heated the chip base to 65 °C. The base then drove the chip to rotate at a low speed of 1600 rpm for 30 s. This process was performed three times to eliminate bubbles and evenly mix the liquid in the sample chamber into the distribution tank. After that, the chip was quickly centrifuged three times at 4500 rpm, each lasting 30 s. Finally, the liquid was centrifuged into the reaction chamber, mixed with primers, and incubated at 65 °C for 40 min. The chip was rotated once every minute, and the fluorescent signals were read during the rotation.

### 2.3. Primer Screening

Because A/H1N1 and B/Victoria had multiple target sites, influenza virus RNA from different regions and years were pooled together as a positive control, while RNase-free water was used as a negative control. For A/H3N2, which had only two target sites for screening, RNA samples from different years were used to evaluate the universality of the inner and outer primers across various epidemic years. LAMP primers targeting different sites were pre-embedded into the reaction chambers of the LAMP microfluidic chip. Among all amplification results, the optimal combination of LAMP inner and outer primers was identified by screening for those exhibiting a typical S-shaped amplification curve. The selected primer sets had the smallest Dt value (threshold time), no threshold signal during the baseline phase, and a stable curve during the plateau phase. The loop primers were designed based on the selected primer sets. The evaluation method for loop primers was consistent with that used for screening the inner and outer primers.

### 2.4. Amplification Using LAMP Microfluidic Chips

The primers required for amplification were pre-embedded into the reaction chambers of the microfluidic chip. After attaching the bottom membrane, the chip was sealed, dried, then stored at −20 °C. Before amplification, warm the chip at room temperature for 10 min and then unpack it for use. A total of 5 μL of the RNA sample was added to 25 μL of the LAMP detection solution (KY0000001, Shanghai Igenetec Diagnostics Co., Ltd.), which was then vortexed and briefly centrifuged (Minispin F-45-12-11, Eppendorf, Hamburg, Germany) to remove bubbles. The entire 30 μL of solution was injected into the sample chambers of the chip via a loading well. After attaching the aluminum top membrane (SC-1003, Shanghai Suxin Biotechnology Co., Ltd., Shanghai, China), a scraper was used to compact and remove air between the chip and membrane. The loaded chip was placed into an automatic isothermal NA amplification analyzer (MA3000, Shanghai Igenetec Diagnostics Co., Ltd.), where the amplification reaction could be initiated with the corresponding sample mode.

### 2.5. Methodological Evaluation

The performance of the selected primers was evaluated in terms of specificity, sensitivity, and reproducibility. The specificity test was designed to exclude the possibility of cross-amplification. Specificity was assessed using total NAs extracted from the swab samples of three common respiratory viruses: respiratory syncytial virus A (RSV-A), respiratory syncytial virus B (RSV-B), and COVID-19, as outgroup samples. Ingroup samples consisted of total NAs from A/H1N1, A/H3N2, and B/Victoria viral cultures. Negative controls consisted of total NAs extracted from healthy human swab samples, with RNase-free water serving as the blank control.

The total NAs of the three influenza viruses were gradient diluted for sensitivity assessment to determine the minimum detectable concentration in actual samples.

The reproducibility assessment consists of two parts: intra-day and inter-day evaluations. Since the microfluidic chip is single use, the intra-day test was conducted on the same chip, while the inter-day test was performed on different chips using the same RNA sample for both tests. The intra-day test data can evaluate the differences between different reaction wells on the chip, while the inter-day test data provides an evaluation of the robustness between chip batches. The Rs calculation for the Dt value is as follows:

(1) $$RSD\%=\frac{Standard\;Deviation}{Mean}\times 100\%$$

### 2.6. Comparison of Actual Sample Detection

Swabs (stored at −80 °C) from patients with A/H1N1, A/H3N2, and B/Victoria (including cases of co-infection with 2–3 of these strains) were collected from various districts in Shanghai over different years. The total NAs extracted from these samples were amplified and detected using both the LAMP microfluidic chip and qPCR methods, and the threshold times (Dt and Cq) from both methods were compared. During LAMP microfluidic chip detection, the threshold time is represented by the Dt value, which indicates the time taken for the fluorescence intensity to exceed the fluorescence threshold and is compared with the Cq value obtained from qPCR results. For A/H1N1 and B/Victoria, qPCR amplification was performed using the Influenza A/B Virus Nucleic Acid Detection Kit (JC10202N, Jiangsu Bioperfectus Technologies Co., Ltd.), while for A/H3N2, the H3N2 Influenza Virus Nucleic Acid Detection Kit (YJC10211, Jiangsu Bioperfectus Technologies Co., Ltd.) was used as a substitute.

The recall rate (Formula (2)) and false-negative rate (Formula (3)) of the LAMP microfluidic chip method in this study were evaluated using the qPCR results as a reference.

(2) $$Recall\;Rate=\frac{True\;Positives}{Actual\;Positives}\times 100\%$$

*Actual Positives* represents the number of samples with positive qPCR results.

(3) $$False\;Negative\;Rate=\frac{False\;Negatives}{Ture\;Negatives+False\;Negatives}$$

## 3. Results

### 3.1. Detection Principle of LAMP Microfluidic Chips

**Figure 1:**
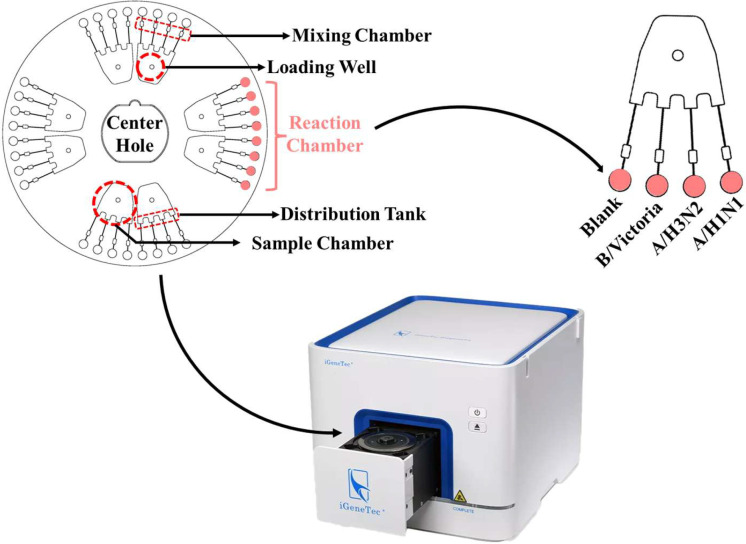
Detection principle of LAMP Microfluidic Chips.

The LAMP microfluidic chip is an updated version from the previous generation of microfluidic chips [29,30], and they share the same principles. As shown in Figure 1, the LAMP microfluidic chip has four independent sectors, and each sector contains two independent detection units. The entire chip can be fixed on a rotating and heated base in the detection instrument through the center hole. The LAMP reaction system containing the RNA to be tested can be injected into the chip through the small hole on the top of the sample chamber. The chip will be centrifuged at a low speed to eliminate bubbles in the sample chamber; preliminarily mix the liquid and allow it to reach the distribution tank. In the subsequent high-speed centrifugation process, the liquid quickly passes through the channel connecting the sample chamber and the reaction chamber by centrifugation. In this process, the liquid quickly mixes the RNA and reagents in the mixing chamber. Finally, 5 μL of liquid fills each reaction chamber and is amplified at 65 °C. Excess reagents, as well as liquid that expands out of the reaction chamber during the reaction, will return to the mixing chamber. The chip was rotated once every minute, and the fluorescent signals from the reaction chambers were read during the rotation.

### 3.2. Primer Sets Screening for LAMP Microfluidic Chips

Multiple sets of primers were designed targeting different sites of the genomes of three influenza viruses to select the most suitable primers for the LAMP reaction.

**Figure 2:**
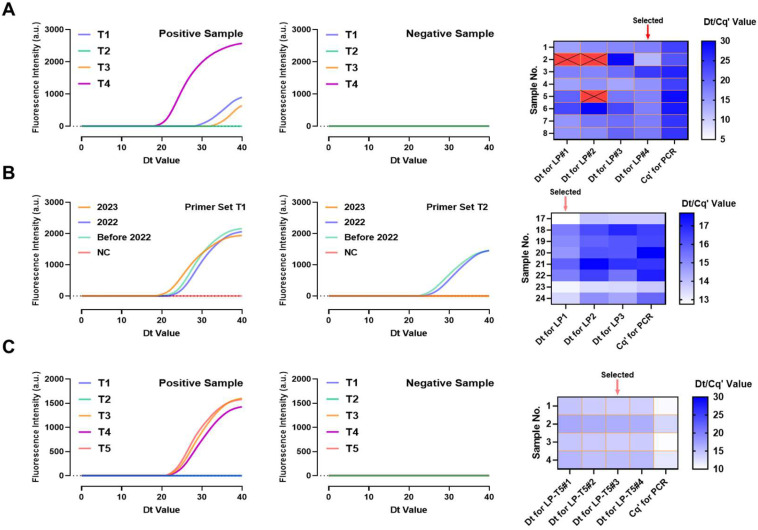
Screening of inner and outer primers and loop primers for A/H1N1 (**A**), A/H3N2 (**B**), and B/Victoria (**C**) using positive and negative controls and comparison with qPCR method. Red blocks represent false negatives.

Four inner and outer primer sets targeting different regions of the A/H1N1 were designed, named T1, T2, T3, and T4, and validated using the same A/H1N1 samples. The T4 primer sets, which showed the smallest Dt value, were selected (Figure 2A). Subsequently, eight different A/H1N1 samples were used to screen for loop primers corresponding to the T4 region. Loop primer LP#4 can efficiently amplify the targets in all eight samples and reduce the runtime by 1.06% compared to qPCR. Primers LP#1 and LP#2 failed to amplify some A/H1N1 samples, while LP#3 had a longer amplification time than qPCR (Table S1) and were thus discarded.

With the same method, inner and outer primers as well as loop primers for A/H3N2 (Figure 2B) and B/Victoria (Figure 2C) were evaluated, resulting in the selection of A/H3N2-T1-LP#1 and B/Victoria-T5-LP#3. The A/H3N2-T1-LP#1 primer set reduced the amplification time by 9.14% (Table S2), while Victoria-T5-LP#3 was the fastest amplification primer set among all B/Victoria loop primers (Table S3).

When screening the A/H3N2 primers, NAs from A/H3N2 of three different years were used to determine whether these primers were universally applicable to strains circulating in different years, as these strains may have mutations. As shown in Figure 2B, the LAMP primers targeting the T1 site were able to amplify a greater number of strains from different years, while primers designed based on the T2 site could not efficiently amplify the A/H3N2 strain circulating in 2023.

### 3.3. Evaluation of Specificity

**Figure 3:**
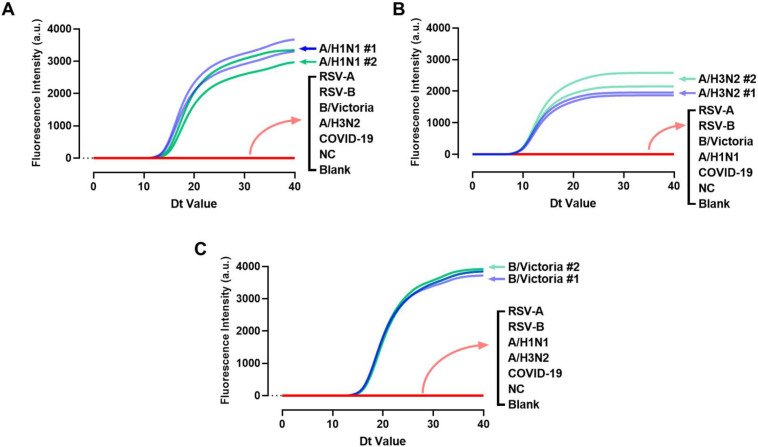
Evaluation of the specificity. (**A**) A/H1N1; (**B**) A/H3N2; and (**C**) B/Victoria.

The LAMP primer sets selected through screening were tested using undiluted total NA from RSV-A, RSV-B, and COVID-19, in addition to A/H1N1, A/H3N2, and B/Victoria influenza viruses. Two different swab samples from each kind of virus were tested. Negative controls were total NAs extracted from the swab samples of healthy humans, and RNase-free water was used as a blank control. The three LAMP primers were able to correctly amplify the corresponding viral samples with no fluorescence signal detected for other viruses or control groups. This confirmed that the primers did not cross-amplify, ensuring good specificity. See Figure 3.

### 3.4. Evaluation of Sensitivity

**Figure 4:**
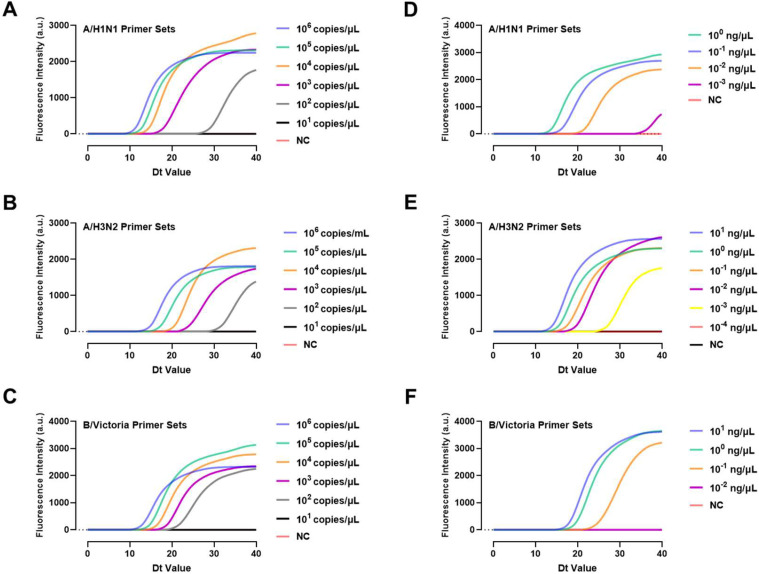
The limit of detection for targets from (**A**) A/H1N1, (**B**) A/H3N2, and (**C**) B/Victoria recombinant plasmid. And the minimum detectable concentrations for total NA from (**D**) A/H1N1, (**E**) A/H3N2, and (**F**) B/Victoria infected swab samples.

After cloning the target fragments corresponding to the LAMP primers for three influenza viruses into plasmids, the copy concentrations were diluted ten-fold to assess the actual sensitivity of the three primer sets. The minimum detectable concentrations for A/H1N1, A/H3N2, and B/Victoria were 10^2^ copies/μL (Figure 4), indicating good sensitivity.

Viral NA samples from patients were then used to evaluate the detection limit under actual testing conditions. Figure 4D–F shows that A/H1N1 and A/H3N2 NAs could be detected at a minimum concentration of 10^−3^ ng/μL, while B/Victoria NA could be detected at 10^−1^ ng/μL.

### 3.5. Evaluation of Reproducibility

Viral NA samples close to the detection limit (with three to four consecutive gradient concentrations) were used to evaluate the repeatability performance of the three primer sets. For A/H1N1, the concentrations of 10^0^, 10^−1^, and 10^−2^ ng/μL were used; for A/H3N2, 10^1^, 10^0^, 10^−1^, and 10^−2^ ng/μL were used; and for B/Victoria, 10^1^, 10^0^, and 10^−1^ ng/μL were used. The deep-colored solid lines represent the average fitting curve of fluorescence signal intensity at the same time for the three repeated experiments, while the corresponding light-colored translucent area indicates the error band of the average fitting curve.

**Figure 5:**
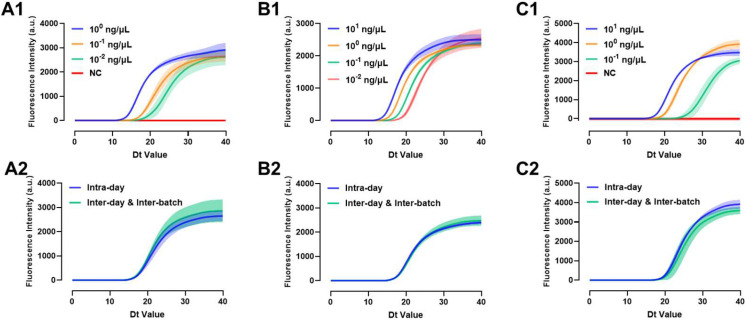
Evaluation of the robustness of Dt values under different concentrations and batches. (**A1**,**A2**) A/H1N1, (**B1**,**B2**) A/H3N2, and (**C1**,**C2**) B/Victoria.

For A/H1N1 and B/Victoria, the error band of the amplification curve gradually widens as the concentration approaches the detection limit in the geometric amplification phase, indicating that the variation in the Dt values of the same concentration NA samples increases. The fluorescence intensity fluctuation range during the plateau phase remains similar (Figure 5(A1,C1)). For A/H3N2, the error band remains narrow in the exponential amplification phase. But when detecting the 10^−2^ ng/μL NA sample, which is close to the detection limit, the error band in the plateau phase widens, indicating that the fluorescence signal intensity fluctuates more, and the resulting amplification product quantity varies significantly (Figure 5(B1)). Tables S4–S6 show that the relative standard deviation (RSD) of the Dt values at concentrations above the detection limit is below 5%, indicating good repeatability.

Inter-batch robustness reflects both primer performance and post-storage stability of the microfluidic chips. Chips with primers from different batches were used to detect the same Viral NA sample. The RSD for Dt values of A/H1N1 and A/H3N2 were below 5%, indicating good stability. The RSD for B/Victoria was 5.623%, showing repeatability (Table S8). Additionally, the error bands for inter-batch robustness were wider than those for intra-day robustness. For A/H1N1 and A/H3N2, the error band variation primarily occurred in the plateau phase (Figure 5(A2,B2)), indicating differences in the final product yield but with consistent Dt values and amplification efficiency. For B/Victoria, the error band differences appeared in the amplification phase (Figure 5(C2)), suggesting variations in Dt values, possibly due to instability in primer amplification efficiency.

### 3.6. LAMP Microfluidic Chip for Complex Virus Sample Detection

**Figure 6:**
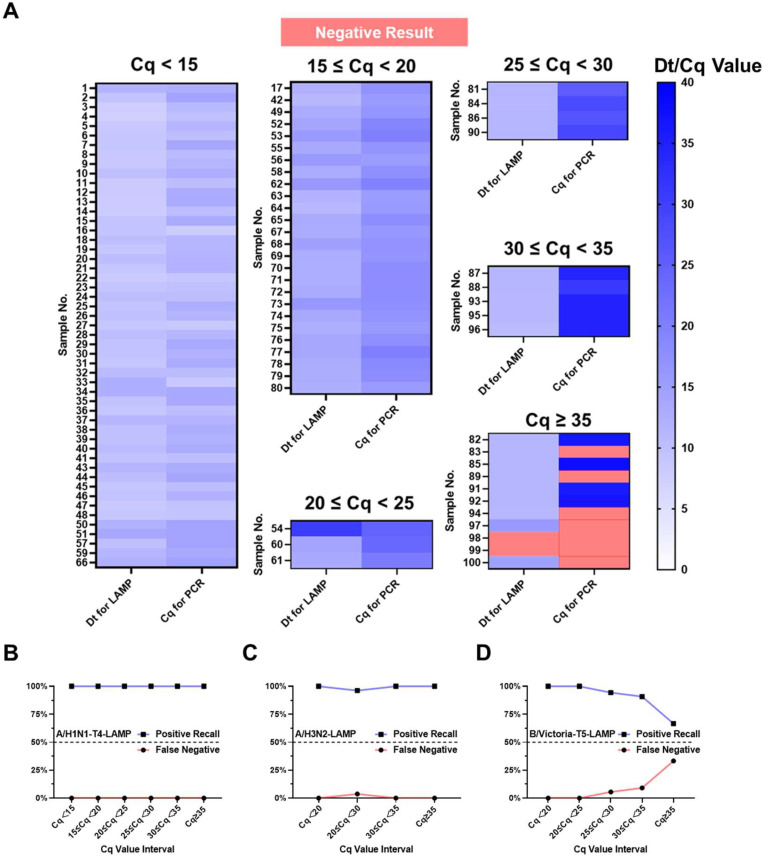
The performance of the microfluidic chip in detection of complex virus sample. (**A**) Detection results of LAMP microfluidic chip method for 100 human samples with A/H1N1 infection, multiple infections, or no infection (n = 2); positive recall and false-positive rate of (**B**) A/H1N1; (**C**) A/H3N2; and (**D**) B/Victoria. Red block represents negative result.

In practical testing, complex samples such as those with multiple infections, improper storage, or NA degradation can complicate detection. This study evaluated the performance of different LAMP primer sets using swab samples from various regions and time points. The influenza strains tested were also assessed using qPCR. For example, in the case of A/H1N1 (Figure 6A), 100 nasal swab samples from patients suspected of A/H1N1 influenza infection, collected from various areas of Shanghai over multiple years, were extracted for total NAs. Each sample was tested using both the LAMP microfluidic chip and qPCR methods, and the resulting Dt and Cq values were presented in heatmaps. The Dt values (left column) were generally lower than the corresponding Cq values, except for the negative samples (red blocks) and sample #54, as evidenced by the lighter color in the left column. This indicates that the A/H1N1-T4 primer set consistently demonstrated a faster detection compared to qPCR, particularly in low-concentration NA targets. As the Cq value increased, the color difference between the Dt and Cq blocks became more pronounced. The Cq blocks darkened significantly, indicating a decrease in sample concentration. Although the Dt block color also darkened with the decrease in sample concentration, it remained notably lighter than the Cq color when Cq ≥ 35, highlighting the advantage of the LAMP primer set in detecting low-concentration NAs. These findings were further validated by results from over hundreds of samples of A/H3N2 and B/Victoria (Figures S1 and S2).

The reliability of the three LAMP primer sets in detecting actual samples on the microfluidic chip was evaluated using the recall rate and false-negative rate for a large number of samples. A recall rate close to 100% and a false-negative rate close to 0% are ideal. In the case of 100 A/H1N1 samples, both metrics were optimal, with the recall rate remaining at 100% across all Cq intervals and the false-negative rate at 0% (Figure 6B). For A/H3N2, the recall rate slightly decreased to 96.3% within the Cq range ∈ [20, 30), with a false-negative rate of 3.7% (Figure 6C). For B/Victoria, both the recall rate and false-negative rate worsened with increasing Cq values. The recall rate dropped to 90.91% within the [30, 35) Cq range and rapidly decreased to 66.67% when Cq ≥ 35 (Figure 6D). This suggests that B/Victoria detection is more reliable when Cq < 35.

### 3.7. Simultaneous Detection

**Figure 7:**
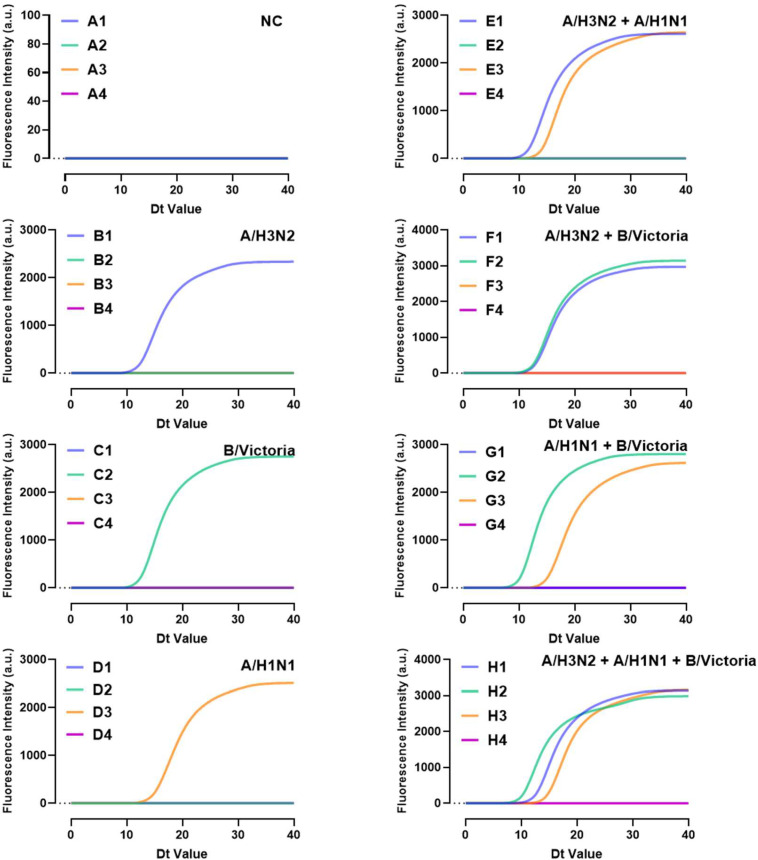
Performance of microfluidic chips in detecting mixed NA samples. Letters represent different sectors on the chip, and numbers 1–4 represent the reaction chambers from right to left.

The eight-sample type LAMP microfluidic chip consists of four sectors, each capable of independently detecting the presence of four pathogens in two NA samples. LAMP primers for the three influenza viruses were embedded into the first three reaction chambers corresponding to a sample well, while the final chamber contained no primers as a blank or reserve (Figure 1). The detection capability of these three primers was evaluated on the eight-sample type chip using the three influenza viruses and their combinations, with each detection chamber correctly identifying the presence of a single virus or their combinations (Figure 7).

## 4. Discussion

A/H1N1, A/H3N2, and B/Victoria are three common influenza viruses with rapid mutation and wide transmission capabilities, creating an urgent need for their rapid detection. We designed and screened highly specific LAMP primers for these three viruses, utilizing a microfluidic chip to achieve simultaneous detection within 1 h. This is similar to the time taken by Oh et al., who also used centrifugal microfluidic chips to detect foodborne pathogens [31]. Compared with the traditional RT-PCR method (90 min of amplification time) [32], the LAMP microfluidic chip can be at least 30 min faster. The classic qPCR method requires multiple wells in a 96-well plate to detect different pathogens, and each pathogen needs to be replaced with the corresponding primer and loaded separately. In contrast, the LAMP-based microfluidic method can detect multiple pathogens simultaneously with only one sample loading step, which is more efficient. A/H1N1 and A/H3N2 can be detected at a minimum NA concentration of 10^−3^ ng/μL, while B/Victoria can be detected at 10^−1^ ng/μL, which achieves a similar sensitivity to other NAAT (NA Amplification Test) methods [33]. At the same NA concentration, the Dt values of the LAMP microfluidic chip show good repeatability. In actual sample detection, when compared to qPCR methods, the positive recall rate is over 90% when the Dt value is less than 35. Ultimately, the validated primers were pre-embedded into an eight-sample type LAMP microfluidic chip, enabling accurate detection in clinical environments with aerosol contamination. Our detection method only requires sample loading and sticker application. The process is simple and aligns well with the integration, convenience, and on-site operability required for POCT.

## Data Availability

The raw data supporting the conclusions of this article will be made available by the authors on request.

## Supplementary Materials

The following supporting information can be downloaded at: https://www.mdpi.com/article/10.3390/bios15080506/s1, Table S1: Evaluation of the A/H1N1 LAMP Loop Primer Set T4 (n = 2). Table S2: Evaluation of the A/H3N2 LAMP Loop Primer Set T1 (n = 2). Table S3: Evaluation of the B/Victoria LAMP Loop Primer Set T5 (n = 2). Table S4: Repeatability of H1-T4 microfluidic LAMP method at different detection concentrations (n = 3). Table S5: Repeatability of H3 microfluidic LAMP method at different detection concentrations (n = 3). Table S6: Repeatability of BV-T5 microfluidic LAMP method at different detection concentrations (n = 3). Table S7: Selected LAMP Primer Sets. Table S8: Robustness of LAMP Amplification Results. Figure S1: Detection results of LAMP microfluidic chip method for 112 human samples with H3N2 infection, multiple infection, or no infection (n = 2). Figure S2: Detection results of T5-LAMP microfluidic chip method for 96 human samples with BV infection, multiple infection, or no infection (n = 2).

## Abbreviations

The following abbreviations are used in this manuscript:

LAMP	Loop-mediated isothermal amplification
A/H1N1	Influenza A H1N1 virus
A/H3N2	Influenza A H3N2 virus
B/Victoria	Influenza B Victoria virus
RSD	Relative standard deviation
NAAT	Nucleic acid amplification test
NA	Nucleic acid
POCT	Point-of-care testing
