# Correction: Zhao et al. LAMP-Based 4-Channel Microfluidic Chip for POCT Detection of Influenza A H1N1, H3N2, and Influenza B Victoria Viruses. *Biosensors* 2025, *15*, 506

**DOI:** 10.3390/bios15110748

**Published:** 2025-11-07

**Authors:** Xue Zhao, Jiale Gao, Yijing Gu, Zheng Teng, Xi Zhang, Huanyu Wu, Xin Chen, Min Chen, Jilie Kong

**Affiliations:** 1 Virus Testing Laboratory, Pathogen Testing Center, Shanghai Municipal Center for Disease Control and Prevention, Shanghai 201100, China; 2 School of Life Science and Technology, Tongji University, Shanghai 200092, China; 3 Chemistry Department, Fudan University, Shanghai 200433, China

## Missing Institutional Review Board Statement

In the original publication [1], the Institutional Review Board Statement and Informed Consent Statement need to be updated as below.

**Institutional Review Board Statement:** The authors state that the study was conducted in accordance with the Declaration of Helsinki and approved by the Ethical Review Committee of Shanghai Municipal Center for Disease Control and Prevention [KY-2024-15] on 22 March 2024.
**Informed Consent Statement:** Patient consent was waived due to the fact that this study does not involve direct patient contact. All samples were collected through the routine influenza surveillance program of the Shanghai Municipal Influenza Surveillance and Prevention for Respiratory Infectious Diseases. No patient privacy was involved, and no personal information such as name, age, or gender was collected. Furthermore, this study is not a clinical report and does not involve any commercial interests.

The authors also state that the scientific conclusions are unaffected. This correction was approved by the Academic Editor. The original publication has also been updated.
