# Supplementary material for: LAMP-Based 4-Channel Microfluidic Chip for POCT Detection of Influenza A H1N1, H3N2, and Influenza B Victoria Viruses
Source: Biosensors (Basel). 2025 Aug 4;15(8):506. doi: 10.3390/bios15080506 (PMC12385032; doi:10.3390/bios15080506)
Supplement: Supplementary file 1 [file biosensors-15-00506-s001.zip › biosensors-3738645-supplementary.pdf]

Table S1. Evaluation of the A/H1N1 LAMP Loop Primer Set T4 (n=2).

Comparison of relative amplification rates of A/H1N1-T4-LP#1

Sample No.	Cq’	Dt for LP #1		$\overline{Dt}$	Δ
#1	15.74	13.58	15.07	14.33	-9.84%
#2	14.40	---	---	---	---
#3	21.49	17.17	17.57	17.37	-23.72%
#4	17.69	14.71	13.75	14.23	-24.31%
#5	15.75	22.56	18.22	20.39	+22.76%
#6	19.27	17.8	28.12	22.96	+16.07%
#7	18.42	14.70	16.00	15.35	-20.00%
#8	16.53	15.04	15.88	15.46	-6.92%
				**Total Average**	**-6.57%**

Comparison of relative amplification rates of A/H1N1-T4-LP#2

Sample No.	Cq’	Dt for LP #2		$\overline{Dt}$	Δ
#1	15.74	16.04	16.43	16.24	+3.08%
#2	14.40	---	---	---	---
#3	21.49	17.96	15.98	16.97	-26.64%
#4	17.69	15.84	15.52	15.68	-12.82%
#5	15.75	---	---	---	---
#6	19.27	29.43	---	29.43	+34.52%
#7	18.42	17.55	18.76	18.16	-1.43%
#8	16.53	16.44	16.61	16.53	-0.00%
				**Total Average**	**-0.55%**

Comparison of relative amplification rates of A/H1N1-T4-LP#3

Sample No.	Cq’	Dt for LP #3		$\overline{Dt}$	Δ
#1	15.74	16.42	17.09	16.76	+6.09%
#2	14.40	---	29.23	29.23	+50.74%
#3	21.49	20.9	17.54	19.22	-11.81%
#4	17.69	13.89	13.6	13.75	-28.65%
#5	15.75	---	18.28	18.28	+13.84%
#6	19.27	22.98	---	22.98	+16.14%
#7	18.42	19.9	18.57	19.24	+4.26%
#8	16.53	18.28	21.61	19.95	+17.14%
				**Total Average**	**+8.47%**

Comparison of relative amplification rates of A/H1N1-T4-LP#4

Sample No.	Cq’	Dt for LP #4		$\overline{Dt}$	Δ
#1	15.74	18.84	16.39	17.62	+10.64%
#2	14.40	12.17	11.98	12.08	-19.25%
#3	21.49	17.17	31.56	24.37	+11.80%
#4	17.69	15.53	15.82	15.68	-12.85%
#5	15.75	21.93	12.30	17.12	+7.98%
#6	19.27	12.81	21.92	17.37	-10.97%
#7	18.42	20.00	15.40	17.70	-4.07%
#8	16.53	15.97	20.07	18.02	+8.27%
				**Total Average**	**-1.06%**

Table S2. Evaluation of the A/H3N2 LAMP Loop Primer Set T1 (n=2).

Comparison of relative amplification rates of A/H3N2-T1-LP#1

Sample No.	Cq’	Dt for LP #1			$\overline{Dt}$	Δ
#17	13.77	---	13.07	12.44	12.76	-7.35%
#18	16.45	14.58	15.87	15.26	15.24	-7.39%
#19	16.32	15.01	15.18	14.46	14.88	-8.80%
#20	17.69	14.43	14.8	15.91	15.05	-14.93%
#21	16.65	15.41	15.8	16.47	15.89	-4.56%
#22	17.09	15.24	15.46	14.72	15.14	-11.43%
#23	13.79	12.63	12.93	13.15	12.90	-6.41%
#24	15.67	13.54	14.19	13.52	13.75	-12.23%
**Total Average**						**-9.14%**

Comparison of relative amplification rates of A/H3N2-T1-LP#2

Sample No.	Cq’	Dt for LP #2			$\overline{Dt}$	Δ
#17	13.77	---	14.23	13.63	13.93	+1.19%
#18	16.45	15.26	16.24	16.25	15.92	-3.26%
#19	16.32	15.52	15.93	15.78	15.74	-3.53%
#20	17.69	15.08	16.07	16.12	15.76	-10.91%
#21	16.65	17.22	17.61	18.33	17.72	+6.41%
#22	17.09	16.46	15.58	16.94	16.33	-4.49%
#23	13.79	13.22	13.43	13.97	13.54	-1.79%
#24	15.67	14.47	15.25	14.91	14.88	-5.04%
**Total Average**						**-2.68%**

Comparison of relative amplification rates of A/H3N2-T1-LP#3

Sample No.	Cq’	Dt for LP #3			$\overline{Dt}$	Δ
#17	13.77	13.46	14.46	13.78	13.90	+0.97%
#18	16.45	15.49	17.92	16.96	16.79	+2.05%
#19	16.32	15.61	16.01	16.21	15.94	-2.31%
#20	17.69	14.75	15.99	17.38	16.04	-9.31%
#21	16.65	16.74	16.71	19.21	17.55	+5.40%
#22	17.09	15.44	16.09	15.35	15.63	-8.58%
#23	13.79	13.1	13.71	13.52	13.44	-2.49%
#24	15.67	14.64	14.48	14.3	14.47	-7.62%
**Total Average**						**-2.74%**

Table S3. Evaluation of the B/Victoria LAMP Loop Primer Set T5 (n=2).

Comparison of relative amplification rates of B/Victoria-T5-LP#1

Sample No.	Cq’	Dt for LP #1		$\overline{Dt}$	Δ
#1	10.41	14.3	14.87	14.59	+40.15%
#2	13.18	16.75	16.81	16.78	+27.31%
#3	10.15	14.44	14.42	14.43	+42.12%
#4	11.81	15.72	15.45	15.59	+31.93%
**Total Average**					**+35.38%**

Comparison of relative amplification rates of B/Victoria-T5-LP#2

Sample No.	Cq’	Dt for LP #2		$\overline{Dt}$	Δ
#1	10.41	13.88	14.01	13.95	+34.00%
#2	13.18	16.65	16.2	16.43	+24.62%
#3	10.15	14.07	14.33	14.20	+39.86%
#4	11.81	15.1	14.75	14.93	+26.34%
**Total Average**					**+31.20%**

Comparison of relative amplification rates of B/Victoria-T5-LP#3

Sample No.	Cq'	Dt for LP #3		$\overline{Dt}$	Δ
#1	10.41	13.79	13.75	13.77	+32.32%
#2	13.18	16.25	16.24	16.25	+23.25%
#3	10.15	13.93	13.81	13.87	+36.61%
#4	11.81	15.1	15.03	15.07	+27.53%
				**Total Average**	**+29.93%**

Comparison of relative amplification rates of B/Victoria-T5-LP#4

Sample No.	Cq'	Dt for LP #4		$\overline{Dt}$	Δ
#1	10.41	13.72	13.71	13.72	+31.79%
#2	13.18	16.07	16.36	16.22	+23.03%
#3	10.15	14.06	13.91	13.99	+37.74%
#4	11.81	15.84	14.9	15.37	+30.11%
				**Total Average**	**+30.67%**

Table S4. Repeatability of H1-T4 microfluidic LAMP method at different detection concentrations (n=3).

Concentration	Robustness		Dt		RSD%
10^0 ng/μL		13.99	13.65	13.76	1.026%
10^{-1} ng/μL	Intra-day	17.04	17.73	17.13	1.770%
10^{-2} ng/μL		21.64	19.10	18.43	7.010%

Table S5. Repeatability of H3 microfluidic LAMP method at different detection concentrations (n=3).

Concentration	Robustness		Dt		RSD%
10^{-1} ng/μL		17.58	17.58	17.55	0.080%
10^{-2} ng/μL	Intra-day	18.92	19.78	19.50	1.846%
10^{-3} ng/μL		36.81	27.23	32.38	12.18%

Table S6. Repeatability of BV-T5 microfluidic LAMP method at different detection concentrations (n=3).

Concentration	Robustness	Dt			RSD%
10^{1} ng/μL		17.41	17.69	17.5	0.666%
10^{0} ng/μL	Intra-day	19.07	19.78	19.99	2.007%
10^{-1} ng/μL		26.74	24.24	27.29	5.087%

Table S7. Selected LAMP Primer Sets

Influenza Virus	Primer	Sequence
A/H1N1-T4	F3	GCTGTGTTAAAATACAATGGCA
	B3	TAGGGGCCTTCATTTCGA
	FIP	GCACATTCAGACTCTTGTGTCCATAACAGACACTATCAAGAGTTGG
	BIP	TTACCATAATGACCGATGGACCATGATTTGATTATCTTTCCCTTCTC
	BL#4	GACAGGCCTCATACAAAATCTTCA
A/H3N2-T1	F3	TCTATTGGACAATAGTAAAACCG
	B3	TGTTTACATTTTGGAACGGTT
	FIP	CGTATTTTGAAGTAACCCCTAGGAGGGAGACATACTTTTGATTAACAGC
	BIP	AAGTGGGAAAAGCTCAATAATGAGAGAATGCTTCCATTTGGAGTG
	BL#1	CAGACGCACCCATTGGCAAA
B/Victoria-T5	F3	ACCTTCTCCGAGGATACG
	B3	CTGTACAAATGTATGGTACTTCT
	FIP	TAGGGTCCTCCTGGTGCATTTAACATGTCAGGTTGTCAACT
	BIP	TTACCAATGGAAAAGGATTCTTCGCTGTTAATGGATTTGTTGCTGTT
	BL#3	TGGCTTGGGCTGTCCCAAAAAA

Table S8. Robustness of LAMP Amplification Results

Primer	Robustness	Concentration	Dt			RSD%
A/H1N1-T4	Intra-day	10^{-1} ng/μL	17.04	17.73	17.13	1.770%
	Inter-day & Inter-batch		17.37	17.13	16.52	2.104%
A/H3N2-T1	Intra-day	10^{-1} ng/μL	17.58	17.58	17.55	0.080%
	Inter-day & Inter-batch		17.58	17.88	17.85	0.759%
B/Victoria	Intra-day	10^{0} ng/μL	19.07	19.78	19.99	2.007%
	Inter-day & Inter-batch		19.07	20.46	21.89	5.623%

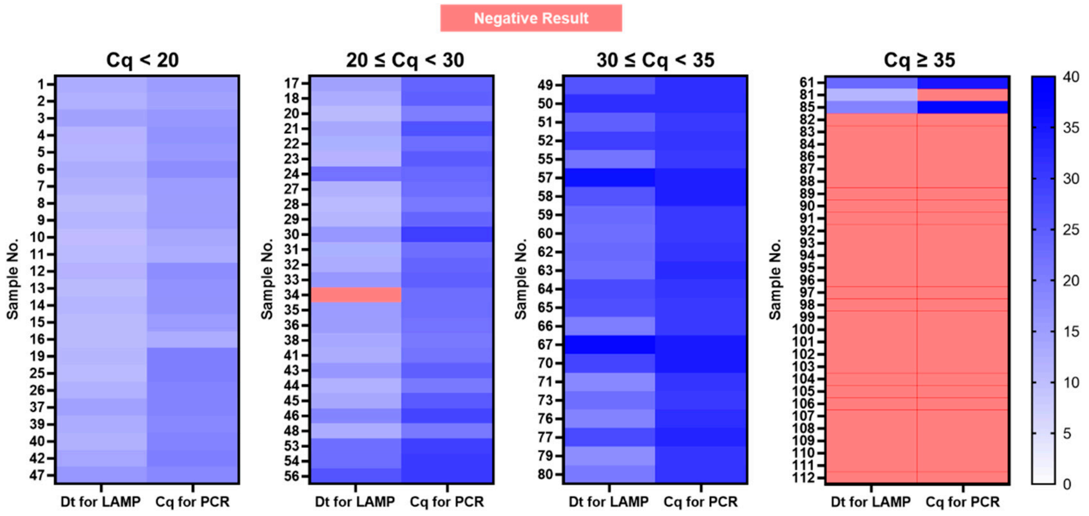

Figure S1. Detection results of LAMP microfluidic chip method for 112 human samples with H3N2 infection, multiple infection, or no infection (n=2).

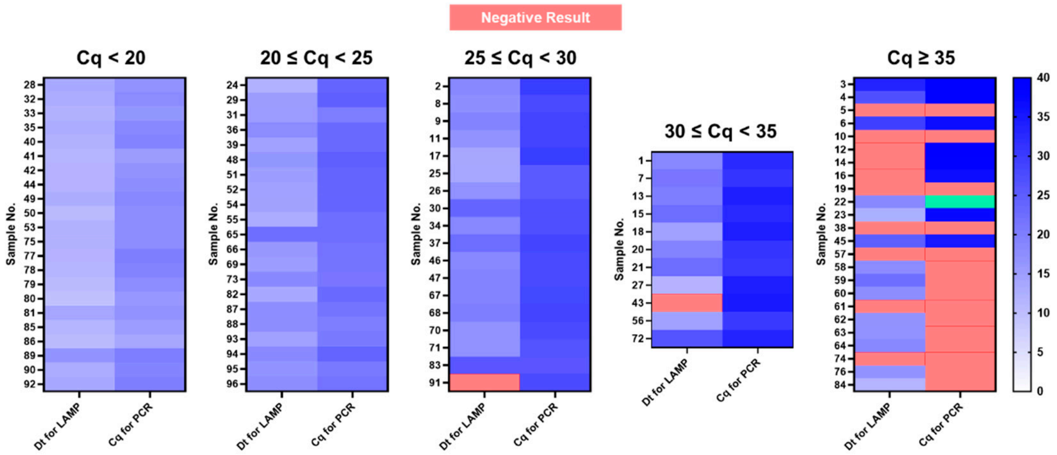

Figure S2. Detection results of T5-LAMP microfluidic chip method for 96 human samples with BV infection, multiple infection, or no infection (n=2).
